# Womens’ experience on barriers and facilitators for implementation with Kangaroo Mother Care at Mbarara Regional Referral Hospital, Uganda: a qualitative study

**DOI:** 10.1080/16549716.2026.2738240

**Published:** 2026-10-01

**Authors:** Santorino Data, Tilda Svensson, Patricia Tushemereirwe, Lea Kreyenbaum, Ashish KC

**Affiliations:** a School of Public Health and Community Medicine, Sahlgrenska Academy, University of Gothenburg, Gothenburg, Sweden; b Department of Paediatrics and Child Health, Mbarara University of Science and Technology, Mbarara, Uganda; c Center for Health and Sustainability, Department of Women’s and Children’s Health, Uppsala University, Uppsala, Sweden

**Keywords:** Health system barrier for KMC, social support for KMC, financial barrier for KMC, health of newborn, motivation for KMC

## Abstract

**Background:**

Kangaroo Mother Care (KMC) is a proven cost-effective intervention for preterm and low‑birth‑weight infants, to provide warmth and secondary health effects. In Uganda, despite a high burden of prematurity, resource constraints limit the delivery of cost-effective interventions.

**Objective(s):**

This study aimed to identify barriers and enablers for implementing KMC, explore mothers’ experience of KMC and apply the i-PARIHS framework to understand why implementation was, or was not, successful at Mbarara Regional Referral Hospital.

**Methods:**

This study was conducted in a rural referral hospital in Uganda with semi‑structured interviews with mothers (*n* = 14) in the KMC ward. Interviews were then translated into English, and the transcripts were subjected to qualitative content analysis using inductive coding. Each code was further grouped into subcategories corresponding to barriers or facilitators. The results were interpreted using the i-PARIHS framework to evaluate why treatment implementation was or was not successful.

**Results:**

Kangaroo Mother Care was found to be largely acceptable and implemented by most participants. Barriers and facilitators were identified within the areas of i) healthcare system, social support, finances, knowledge and beliefs as well as the health of newborns and motivation. Primary barriers included insufficient healthcare facilities, lack of material resources, lack of education on KMC, and lack of adaptation to twins. Facilitators included supportive healthcare staff, learning tools, and the positive effects of KMC as motivation.

**Conclusions:**

Barriers and facilitators for Kangaroo Mother Care are multifaceted. To increase implementation for all patients, targeted interventions may need to reach beyond the hospital, addressing social and financial factors.

## Paper Context

- **Main findings:** KMC implementation in rural Uganda is shaped by multiple interconnected factors across the health system, individual, and socio-economic domains.
- **Added knowledge:** Even though mothers perceive KMC as beneficial, its consistent practice is influenced by access to resources, the quality of education and counselling, financial constraints, and the availability of social support.
- **Global health impact for policy and action:** The interventions to improve adherence to KMC require the engagement of hospital leadership, mothers, and social networks in identifying barriers and using a participatory approach to overcome them.

## Background

Neonatal mortality remains a substantial global health problem, accounting for approximately 2.3 million deaths annually and nearly half of all deaths among children under the age of five [1]. Although the under‑five mortality rate (U5MR) has declined substantially over recent decades, this progress has been unequal in terms of specific age groups. The observed reductions have primarily occurred among children aged 1–59 months, while declines in neonatal mortality have been slower. As a result, the proportion of deaths occurring in the neonatal period is increasing, and global progress is slowing, particularly in low- and middle-income countries [2].

Regional disparities are pronounced, with sub-Saharan Africa bearing the highest burden of neonatal and under-five mortality. The region remains off track to meet Sustainable Development Goal (SDG) targets, with slower declines compared to global averages [2]. Uganda has achieved substantial reductions in U5MR from 146 to 39 deaths per 1000 live births between 2000 and 2023, yet neonatal mortality has declined more modestly, from 32 to 18 per 1000 live births [3]. Current trends indicate that accelerated progress is required to meet SDG targets [2].

Prematurity and low birth weight are the leading risk factors for neonatal mortality and contribute to increased vulnerability to hypothermia, respiratory distress, and infection [4–7]. These conditions underpin many of the most common causes of neonatal death, including complications of prematurity and intrapartum-related events [2]. Facility-based studies in Uganda have identified prematurity, asphyxia, sepsis, and hypothermia as major contributors to neonatal mortality, with several of these being preventable or manageable through improved neonatal care [8,9].

Kangaroo Mother Care (KMC), a low-cost and evidence-based intervention for preterm and low‑birth‑weight infants, involves prolonged skin-to-skin contact, early initiation of breastfeeding, and continued care after discharge [10]. KMC has been shown to reduce neonatal mortality by 32%, as well as decrease hypothermia and severe infections, while improving breastfeeding outcomes and maternal wellbeing [10,11]. Due to its low resource requirements, KMC is particularly suitable for low-resource settings.

Despite its proven effectiveness and inclusion in national guidelines, implementation of KMC in Uganda remains inconsistent [12,13]. Barriers include limited infrastructure, insufficient supplies, inadequate training of healthcare workers, and suboptimal documentation and follow-up [14]. In addition, maternal perceptions and experiences may influence uptake and adherence but remain underexplored.

Understanding both health system factors and maternal perspectives is essential to improving KMC implementation and neonatal outcomes. Therefore, the aim of this study has three aims: first, to identify barriers and facilitators to KMC implementation; second, to explore mothers’ experience of the KMC; and third, to apply the i-PARIHS framework to analyze why the implementation of KMC did or did not succeed.

## Methods

### Study setting

This study was conducted in October and November 2025 at the KMC ward in Mbarara Regional Referral Hospital (MRRH), Uganda. The KMC unit had a dedicated space with two KMC beds.

#### Study design

To analyse the interview transcripts, a qualitative content analysis was employed, using an inductive approach as described by Graneheim and Lundman [15]. To address the primary and secondary aim, the inductive coding process was guided by the three research questions, identifying meaning units corresponding to each question. The meaning units were condensed and ascribed code names. Similar codes were then grouped into subcategories which made up the three analytic domains: meaning barriers, facilitators, and perceptions. The findings were interpreted using the integrated Promoting Action on Research Implementation in Health Services (i-PARIHS) framework to evaluate implementation of KMC [16].

#### Participants and recruitment

Study participants were mothers who had been prescribed KMC and stayed at the KMC ward for a minimum of 5 days. They had to be above 18 years of age and be fluent in English or Runyankore. Patient charts were collected each morning to identify eligible mothers. The inclusion criterion requiring mothers to have kept their newborn in KMC for 5 days was intended to ensure adequate experience with initiation and continuation of KMC. The five-day period was intended to provide adequate experience for mothers for interaction with staff and understanding of the context of the KMC ward in the hospital. If charts were unavailable or lacking documentation, a nurse and research assistant on the ward would read out the eligibility criteria and ask affected mothers to raise their hand. Eligible mothers were thereafter randomly selected. Of the 14 interviews conducted, 2 pilot interviews were carried out to test and refine the interview guide based on participant feedback and revise the probes. These two interviews were not included in the final analysis, and only 12 interviews were coded and analyzed; these are the 12 mothers described in the results section. Due to the constraints of having the transcription of the interview in the local language and translation into English after each interview, the saturation could not be assessed concurrently with data collection, which is the recommended approach. We collected data from 12 participants over a span of 2 months; transcripts were reviewed and analyzed in the order they were collected, and no new ‘codes’ or ‘sub-categorises’ emerged in the final three transcripts, which indicates that sufficient depth and range of information had been reached for the study’s aims.

Due to limited time, saturation was not evaluated continuously during data collection. After data collection and acquiring transcripts, data saturation was considered reached.

#### Data collection

The semi-structured questionnaires were constructed based on research questions. A draft was first developed by the second author (TS) and was reviewed by the first author (DA) and the last author (AKC). The translation of the questionnaires into the local language (Runyankore) and the back translation of the questionnaire were done by the third author (PT). The content validity of the back translated questionnaire and the original questionnaire developed by the second author (TS) was assessed by the fourth author (LK). Once the content validity of aligning the back translated and original questionnaire was completed, the third author conducted the cognitive testing of the final translated questionnaires with two KMC mothers. Based on the cognitive testing of the questionnaires on their understandability, the questionnaires were then finalized for implementation. The mothers who were included in the cognitive testing were not included in the main study sample. Semi-structured interviews using an interview guide were conducted in a secure and neutral location within close reach of the KMC ward. Thirteen of the 14 interviews were conducted in Runyankore, and 1 was conducted in English. Interviews were recorded using a tape recorder and directly transferred to a hard drive for storage. Notes were taken for each interview to provide context for the inductive coding.

#### Tool

The interview guide was formatted with previous similar studies in mind. It was tested twice to ensure understanding and an appropriate sequence between topics. The interview guide was thereafter revised to adjust for repeating questions, to group questions more appropriately, and to add questions about breastfeeding.

#### Interviewer characteristics

The interviewer (PT) was a local, female research nurse experienced in qualitative healthcare research, which helped minimize language barriers, misunderstandings, and cultural barriers. The interviewer did not work in the hospital and had no relationship with the participants.

#### Data analysis

The interviewer transcribed the recordings into English without an intermediate transcription in the local language. The first two transcripts were coded individually by the student and a research assistant and then revised together to resolve discrepancies. The following 10 transcripts were coded by the student with consulting from an experienced qualitative researcher. Coding was conducted manually using Microsoft Word. Similar codes were grouped together, and all codes were divided into 5 categories. Commonly reoccurring codes were then grouped into subcategories corresponding to barrier, facilitator, or perception within each main category. After the subcategories were developed, the result was analysed using the i-PARIHS framework.

#### Ethical approval

Ethics application was approved by the Swedish Ethical Review Authority with approval number 2025–07087-01-884399. Ethics application was also approved by the Ugandan Mbarara University of Science and Technology (MUST) Research Ethics Committee with approval number MUST-2025–507. Anonymity was ensured through removal of personal information; each transcript was provided with a code name. Furthermore, sensitive data, including recordings, were stored on hard drives. Informed consent was obtained using written forms, stating anonymity and the ability to decline answering or withdraw at any time for any reason without the provided care being affected. For one patient who could not read, consent was obtained orally. Concern was taken to recruit before morning rounds, and interview times were flexible depending on the participants needs. To reduce discomfort and unfamiliarity as much as possible, a local female interviewer conducted the interviews. A small gift of soap and diapers were given to each participant per advice about local customs. The study was conducted in accordance with the Declaration of Helsinki.

## Results

### Sample description

Sociodemographic characteristics of the interviewees are summarized in Table 1. Twelve mothers were interviewed. Ages ranged between 18 and 37. The number of previous births ranged between 0 and 5. The majority of participants had completed all 7 years of primary school; however, one participant had completed up to 6 years of education, and one participant had received no formal education. Three participants were not working, three were farmers, two worked in finance and accounting, two were tailors, one was a teacher, and one was a waitress. Mapped onto the i-PARIHS framework, themes 1 and 4 (health system factors and financial constraints) correspond to the context construct, which includes infra-structure, resources, and the socio-economic environment for implementation. Theme 2 (parental experience and motivation) and theme 3 (knowledge and beliefs) correspond to the recipient construct on capacity, motivation, and belief as an adopter of KMC. Theme 5 (social support) provides recipients and context, reflecting the facilitation provided by peers and family members.

**Table 1.** Participant characteristics.

Age in years	Education	Occupation	Marital status	Previous births
35	Tertiary	Not working	Married	3
27	Tertiary	Teacher	Married	0
28	Primary	Tailor	Married	3
37	None	Farmer	Married	5
34	Primary	Farmer	Married	3
22	Primary	Not working	Married	0
21	Tertiary	School bursar	Married	0
22	Secondary	Waitress	Unknown	0
24	Primary	None	Married	1
29	Tertiary	Accountant	Married	0
18	Primary	Tailor	Separated	0
22	Secondary	Not working	Married	0

### Theme 1- – health system factors influence initiation and practice of KMC

Participants’ perceived a strong influence of health system factors on the initiation and continuation of KMC. Barriers were predominantly structural and informational, including insufficient education, limited infrastructure, and a lack of material resources. At the same time, facilitators such as supportive healthcare workers and accessible learning tools partially mitigated these challenges. Notably, participants did not express negative perceptions of KMC itself; rather, difficulties were attributed to the conditions under which KMC was implemented.

#### Lack of KMC information and counselling for high-risk infants

Many participants described receiving little or no instruction on KMC upon transfer to the KMC ward as a barrier. Narratives highlighted a lack of structured introduction, leaving mothers uncertain about how to initiate and perform KMC independently. This gap was particularly evident among mothers of high-risk infants, such as twins, where no tailored guidance was provided.

One participant described being transferred without any explanation of the practice: *‘They took me to the kangaroo mother care ward, showed me the bed for my baby, and then left without providing any further instructions … ’* (IDI_KMC_03).

Similarly, another mother of twins emphasized the absence of practical guidance and the resulting confusion: *‘I was left there without guidance on managing kangaroo care for two babies … I was confused about how to carry both babies on my chest simultaneously.’* (IDI_KMC_09).

These quotes suggest that, while KMC may be institutionally recommended, its implementation is not supported by structured education. This lack of guidance appeared to delay initiation and reduce mothers’ confidence in performing KMC correctly.

#### Insufficient infrastructure for KMC

Participants consistently described infrastructural constraints, as a major barrier to practicing KMC. Overcrowding, inadequate sleeping arrangements, and lack of privacy were recurrent concerns. These conditions not only affected maternal wellbeing but also created perceived risks for the infants.

Several mothers reported being unable to rest adequately due to lack of beds or bedding: *‘We are not allowed to sleep in the ward with our babies … we are forced to sleep on the cold tiles under the baby’s bed … ’* (IDI_participant_01).

Overcrowding was also described as compromising infant safety: *‘Seven babies share one bed, which is risky due to the potential spread of infections and accidents like falls.’* (IDI_participant_07).

In addition, the nature of KMC requiring continuous skin-to-skin contact made lack of privacy particularly challenging: *‘It can be challenging to maintain privacy … you end up exposing your body to others … ’* (IDI_participant_02). These findings indicate that inadequate infrastructure not only limits the feasibility of continuous KMC but may also discourage adherence due to physical discomfort, fatigue, and concerns about dignity and safety.

#### Lack of KMC resources

A further barrier identified was the lack of essential materials required to practice KMC effectively. Participants reported that items such as baby wraps, clothing, diapers, and even basic necessities like food and medications were not consistently provided by the hospital.

As one participant explained: *‘The hospital doesn’t supply these items, so it’s our responsibility to have them ready.’* (IDI_participant_02). This reliance on personal resources places an additional burden on families, particularly in low-resource settings, and may limit the ability of some mothers to practice KMC as recommended. The absence of appropriate materials also affects the quality and safety of KMC, especially when secure positioning of the infant cannot be ensured.

#### Supportive healthcare workers

Despite systemic challenges, many participants highlighted the positive role of healthcare workers in facilitating KMC. Healthcare providers were described as approachable, compassionate, and willing to assist when needed.

One participant noted: *‘They are approachable … and go out of their way to ensure our babies receive the best possible care.’* (IDI_Participant_12). Supportive interactions with healthcare workers appeared to enhance mothers’ confidence and ability to continue KMC, even in the presence of other barriers. However, these facilitatory effects seemed dependent on individual staff engagement rather than consistent system-level support.

#### KMC learning tools

In addition to direct support from healthcare workers, participants identified educational materials such as videos and wall charts as valuable facilitators of KMC.

These tools enabled mothers to learn independently and revisit instructions as needed: ‘*The wall charts … provided step-by-step instructions, and I could learn at my own pace.’* (IDI_Participant_11).

Importantly, these resources also facilitated the involvement of family members: *‘My husband … learned how to do it by watching the videos … ’* (IDI_Participant_02). Such tools appeared to compensate, at least partially, for gaps in formal education and support shared caregiving, which may enhance the sustainability of KMC practice.

### Theme 2- – parental health experiences and perceived benefits shape motivation to practice KMC

Participants highlighted how parental experiences (both physical and emotional) strongly influenced engagement with KMC. Mothers generally perceived KMC as beneficial, describing enhanced bonding and observable improvements in their baby’s health, which served as key motivators for continued practice. However, several barriers emerged, including challenges with exclusive breastfeeding, physical discomfort, and concerns about potential health risks for the infant. Encouragement from healthcare workers further reinforced motivation and adherence.

#### Bonding and emotional fulfilment from KMC

A common perception among participants was that KMC promotes a strong emotional connection between mother and child. Mothers frequently describe feelings of comfort, reassurance, and emotional strength when practicing skin-to-skin care. This sense of closeness appeared to reinforce maternal identity and provided psychological support during a stressful period.

One participant expressed: *‘Carrying my baby on my chest makes me feel happy and reassured that she is safe … it brings me comfort and hope for her survival.’* (IDI_Participant_01).

Another highlighted how physical closeness strengthened maternal attachment: *‘The feeling of my baby’s warmth and closeness brings out the motherhood in me … ’* (IDI_Participant_03). These accounts suggest that KMC not only functions as a clinical intervention but also as an emotionally meaningful practice that enhances maternal wellbeing and resilience.

#### Observable health effects of KMC

In addition to emotional benefits, participants perceived KMC as an effective treatment based on visible improvements in their infants’ condition. Weight gain, improved thermoregulation, and better respiratory function were commonly reported. This helped reinforce confidence in the intervention.

One mother described significant progress over time: *‘He is gaining weight daily … his body temperature has improved … we attribute this progress to kangaroo mother care.’* (IDI_Participant_02).

Similarly, another participant noted: *‘The baby’s breathing has also improved, and he is more active.’* (IDI_Participant_04). These observations appeared to strengthen adherence, as mothers directly associated KMC with positive health outcomes.

#### Lack of exclusive breastfeeding

Despite being a core component of KMC, exclusive breastfeeding was not consistently practiced.

Participants described challenges related to insufficient milk production and the clinical condition of the infant, particularly among those requiring tube feeding. *‘My milk supply was still limited. I continue to supplement with formula.’* (IDI_Participant_06).

Another respondent said: *‘The baby is still too small to breastfeed directly … we’re still feeding through the tube.’* (IDI_Participant_05). These findings suggest that structural and physiological constraints may limit full adherence to KMC protocols, even when mothers are motivated to comply.

#### Discomfort associated with KMC practice

Physical discomfort was another commonly reported barrier, particularly during the initial stages of KMC.

Participants described fatigue and pain associated with prolonged skin-to-skin positioning, which could discourage the continuation. One mother said: *‘The first time was tiring, and I felt pain in my chest … I thought I wouldn’t continue.’* (IDI_Participant_11). Although some mothers adapted over time, initial discomfort appeared to pose a challenge to early adherence.

#### Insecurity regarding potential health risks

Some participants expressed concerns that KMC could negatively affect their infants, particularly in cases involving fragile or unstable conditions. Episodes such as apnea led to fear and uncertainty about the safety of prolonged skin-to-skin contact.

One respondent said: *‘He would stop breathing while on my chest, and it was terrifying … I was hesitant to continue.’* (IDI_Participant_02). These perceptions highlight the importance of reassurance and clinical guidance, as uncertainty about infant safety may undermine confidence in the intervention.

#### Health benefits as motivation

Despite the challenges described, perceived improvements in infant health served as a strong motivator for continued KMC practice. Mothers often expressed determination to continue, even under difficult circumstances, due to the visible benefits for their child.

One mother stated: *‘Even when I’m struggling, I see the positive impact … and that motivates me to keep doing it.’* (IDI_Participant_03). This suggests that visible evidence of effectiveness plays a critical role in sustaining adherence.

#### Encouragement from healthcare workers

Encouragement and reassurance from healthcare workers were frequently described as essential for maintaining motivation. Participants emphasized the importance of regular follow-up, positive reinforcement, and clear communication from the staff.

**One mother said:** *‘They remind us that the more we practice … the sooner we shall be discharged. Their motivation has been instrumental … ’* (IDI_Participant_10). Such support appeared to mitigate doubts and reinforce mothers’ commitment to KMC, particularly when facing physical or emotional challenges.

### Theme 3 – knowledge and beliefs about KMC shape understanding and practice

Participants’ narratives revealed that knowledge and beliefs about KMC varied widely and influenced how the intervention was understood and practiced. While most mothers demonstrated a general awareness of the purpose of KMC, gaps and inconsistencies in knowledge were evident, particularly regarding practical application and recommended routines. Many participants were unfamiliar with KMC prior to admission, and some described tensions between the method and existing cultural norms. Notably, no clear facilitators emerged within this theme. Instead, variability in knowledge and persistent uncertainties shaped mothers’ experiences.

#### Mothers’ understanding of the purpose of KMC

Most participants demonstrated a basic understanding of KMC as a health-promoting intervention for premature or low birth weight infants. The primary perceived benefits included maintaining warmth and promoting weight gain, although some mothers also recognized its role in fostering emotional bonding and psychological wellbeing.

One participant explained: *‘It helps strengthen their bones, provides warmth … promotes growth, and fosters a sense of love and bonding.’* (IDI_Participant_09). These narratives suggest that while biomedical aspects of KMC were relatively well understood, the psychosocial benefits were also acknowledged, which points to a holistic perception of the intervention.

#### Hospital requirements and variation in adherence guidance

Despite similar understandings of KMC’s purpose, participants reported considerable variation in the instructions they received regarding the duration and frequency of practice. Recommendations ranged from short, intermittent sessions to prolonged daily contact, reflecting inconsistency in guidance from healthcare providers.

For example, participants described differing instructions: *‘KMC three times a day … morning, afternoon, and evening … ’* (IDI_Participant_01), *‘30 minutes each … ’* (IDI_Participant_06), *‘1 hour, 4 times a day … ’* (IDI_Participant_10), or *‘8 hours a day, broken into sessions … ’* (IDI_Participant_11). This variability appeared to create uncertainty about what constituted adequate practice and may have contributed to inconsistent adherence. It also suggests a lack of standardized communication regarding KMC protocols in MRRH.

#### Unfamiliarity with the KMC method

A common finding across narratives was that many mothers had no prior knowledge of KMC before hospital admission. Initial exposure to the practice often occurred through observation of other mothers rather than formal instruction.

**One mother said:** *‘When I arrived, I saw women carrying their babies on their chests and asked why … ’* (IDI_Participant_07).

For some participants, KMC also conflicted with established cultural practices, particularly norms around infant carrying. One mother highlighted concern related to cultural expectations for twins: *‘In our African tradition … everything must be done equally … I worry about how to carry both on my chest … ’* (IDI_Participant_09). These accounts indicate that unfamiliarity, combined with cultural considerations, may complicate early acceptance and implementation of KMC.

#### KMC becomes easier with practice

Despite initial uncertainty and difficulty, several participants described how KMC became more manageable over time. Repeated practice increased confidence and competence, enabling mothers to integrate the intervention into their daily routines. One respondent reported: *‘With practice, it has become easier … I can now do kangaroo mother care with both babies simultaneously … ’* (IDI_Participant_10). Some participants also became supportive roles for others: *‘Now I’m even helping new mothers … I’m a “star” in the kangaroo mother care unit now.’* (IDI_Participant_11). These narratives suggest that experiential learning plays a key role in overcoming initial barriers and sustaining KMC practice.

*Knowledge gaps among mothers of twins-* A specific barrier identified within this theme was the lack of tailored information for mothers of twins. Standard KMC guidance was often perceived as designed for single infants, leaving mothers uncertain about how to adapt the practice. One mother said: *‘My main challenge was figuring out how to care for my twins simultaneously … I was stuck and didn’t know what to do.’* (IDI_Participant_09). This gap appeared to increase reliance on informal learning and contributed to confusion and delayed confidence. It highlights the need for more inclusive and context-specific education that addresses diverse caregiving situations.

### Theme 4 – financial constraints limit mothers’ ability to sustain KMC

Participants’ narratives highlighted financial hardship as a significant barrier to the practice of KMC. The economic burden associated with prolonged hospital stays, combined with out-of-pocket expenses for basic needs, created substantial challenges for mothers and their families. These constraints affected not only their ability to remain in the hospital but also their physical wellbeing and capacity to provide continuous care. No facilitators were identified within this theme, underscoring the pervasive impact of financial limitations.

#### Financial loss associated with prolonged hospital stay

Extended hospitalization required for KMC was frequently described as economically hard. Participants reported both direct costs, including purchasing medications and supplies, and indirect costs, including loss of income due to inability to work. In some cases, accompanying family members also had to interrupt employment, which further exacerbated financial strain. One participant described the combined impact of lost income and inability to afford necessities: *‘We are struggling financially … my husband … can’t work because he is always here with me. We have gone for a day and a night without eating anything … ’* (IDI_Participant_01). In addition, participants emphasized that many essential items were not provided by the hospital: *‘We have to buy everything else, including some medications like caffeine … this is a major burden … ’* (IDI_Participant_01). These experiences suggest that the cost of care extends beyond medical treatment, placing considerable pressure on already vulnerable households and potentially limiting the feasibility of prolonged KMC.

#### Lack of food and its impact on caregiving

Food insecurity emerged as a recurring and critical barrier. Participants described how lack of access to adequate nutrition affected both their physical capacity to perform KMC and their psychological wellbeing. Hunger was reported to reduce energy levels, making it difficult to sustain prolonged KMC practice. One mother said: *‘It’s challenging to focus on caring for my baby when I’m hungry.’* (IDI_Participant_03). In some cases, minimal support from the hospital was insufficient to meet nutritional needs: *‘The hospital’s support with flour is helpful, but it’s not enough; I can only afford one meal a day.’* (IDI_Participant_06). Participants also linked inadequate nutrition to reduced breastmilk production, which may further compromise adherence to recommended KMC practices. These findings illustrate how financial constraints and food insecurity are closely interconnected, with direct implications for both maternal wellbeing and infant care.

### Theme 5 – social support networks play a dual role in enabling and constraining KMC

Participants’ narratives highlighted the important role of social support networks in shaping their ability to practice KMC. While support from partners, family members, peers, and the wider community often facilitated KMC through emotional encouragement and also practical assistance, it could also introduce strain. This is particularly true for spouses managing financial and caregiving responsibilities. Despite these challenges, social networks function as a key enabling resource.

#### Spousal emotional and financial strain

Although many participants described their partners as supportive, several expressed concerns about the emotional and financial burden placed on them during prolonged hospital stays. Spouses were often required to balance caregiving responsibilities, financial provision, and household duties, leading to exhaustion and stress. One participant reflected on the toll this had taken: *‘He has been with me throughout this journey, but I can see the toll it’s taking on him … he has used up his savings … ’* (IDI_Participant_01). Another highlighted the redistribution of responsibilities within the household: *‘I have five other children at home … and my husband is shouldering the responsibility alone.’* (IDI_Participant_04). These experiences suggest that while spousal support is critical, it may come at a significant cost that potentially affects the sustainability of support over time.

#### Peer learning and support

Fellow mothers in the KMC ward emerged as an important source of both emotional and practical support. Participants frequently described relying on peers for guidance, reassurance, and shared problem-solving, particularly in the absence of formal instruction. One mother said: *‘Whenever I had a question … I would consult with them, and they would provide guidance and reassurance.’* (IDI_Participant_03). Peer support also enabled mothers to manage daily tasks and rest: *‘They will watch my baby while I wash clothes or take a break.’* (IDI_Participant_01). These interactions suggest that informal peer networks can partially compensate for gaps in healthcare system support, while also promoting a sense of community and shared experience.

#### Community and family support

Beyond the hospital setting, participants described varying degrees of support from family members and their wider community. This support included caregiving for children at home, provision of food and resources, emotional encouragement, and direct involvement in KMC practice. One mother stated: *‘My neighbor has been helpful in looking after our other children … ’* (IDI_Participant_01). Some participants described active involvement of partners in KMC: *‘He has been very supportive, and we alternate shifts in caring for the baby like feeding, burping, and doing kangaroo mother care. At night, I breastfeed, and he takes care of the rest, including kangaroo care and monitoring the baby while I rest.’* (IDI_Participant_02). Extended family members also played a crucial role: *‘My mother … has been a lifesaver … she fetches food, does laundry, and keeps the baby when I need to rest.’* (IDI_Participant_03). In addition, emotional encouragement from friends contributed to sustaining motivation: *‘My friends have also been supportive, visiting us at the hospital, encouraging me, and telling me not to give up.’* (IDI_Participant_07). These findings indicate that strong social networks can significantly enhance mothers’ ability to engage in KMC by reducing practical burdens and reinforcing emotional resilience.

## Discussion

This study identified multiple factors influencing the implementation of KMC across health system, individual, and socio-economic levels. Mothers generally perceived KMC as beneficial, citing improved infant health and strengthened bonding as key motivators for continued practice. However, important barriers were identified. At the health system level, insufficient education, inconsistent guidance, inadequate infrastructure, and lack of resources limited optimal practice. At the individual level, physical discomfort, feeding challenges, and concerns about infant safety affected mothers’ ability to sustain KMC. Financial constraints, including loss of income and food insecurity during prolonged hospital stays, further restricted adherence. Social support played a dual role: peer and family support facilitated KMC, while increased strain on spouses highlighted underlying economic pressures. Overall, while acceptability of KMC was high, its implementation was constrained by structural and contextual challenges.

Using the i-PARIHS framework, the results suggest that **innovation**, KMC was well received by mothers; however, negative perception centered on the implementation of the intervention. The **recipients**, (mothers and family members) were motivated due to increased bonding with the newborn despite discomfort. The **context** of health system constraints on staffing and infra-structure for implementing KMC was explained by mothers. Continuous **facilitation** from the hospital staff was a constraint; however, the social support from family members and peers was valuable in facilitating KMC implementation for mothers.

These findings show considerable overlap with several international and regional studies on the implementation and perception of KMC in low-resource settings, while also showing context-specific differences. Consistent with studies from sub-Saharan Africa, KMC was widely perceived by mothers as a beneficial tool, particularly in terms of improving neonatal health and strengthening maternal-infant bonding. Similar perceptions were described in eastern Uganda, where mothers described KMC as essential for warmth, growth and bonding [17]. This reinforces/underscores the point that KMC is highly accepted across several settings, even in the light of challenges. The health system barriers identified in our study closely mirror findings from Côte d’Ivoire and Ethiopia. In Côte d’Ivoire, Kourouma et al. reported lack of space, supplies, and human resources as key barriers to early KMC implementation [18]. Bilal et al. similarly highlighted shortages of supplies and variability in healthcare worker engagement at facility level in Ethiopia [19]. Additionally, the inconsistency in KMC guidance and instructions observed in our study also aligns with these studies [18], suggesting that lack of standardized training and communication remains a widespread challenge.

Socioeconomic constraints, including food security and financial strain due to prolonged hospital stays, were also prominent in our study and align closely with the existing literature. Kourouma and colleagues [18] identified a lack of food for mothers as a critical factor, while Bilal et al. emphasized the broader influence of socioeconomic factors on KMC uptake [19]. Together, these findings underscore the importance of addressing financial and nutritional needs as part of KMC implementation strategies, rather than viewing the intervention solely as a low-cost clinical solution.

At the individual level, our study identified physical discomfort, fatigue, and concerns about infant safety as barriers to sustained KMC practice. Similar challenges were reported by Naloli et al., where maternal stress and physical health issues, including fatigue and pain, limited adherence [17]. Additionally, fear of harming fragile infants, particularly among caregivers and healthcare providers, has been documented in Uganda [20], which closely reflects the concerns expressed by participants in our study regarding apnea and infant vulnerability. These parallels suggest that both physical and psychological burdens are common barriers across contexts.

The role of social support networks in facilitating KMC was another area of strong convergence. Peer support within the ward and the involvement of family members were key facilitators in our study, consistent with findings from both Uganda and Ethiopia [19,20]. Naloli et al. emphasized the importance of family support and substitute caregivers [17], while Tumukunde et al. identified the presence of a family member in the hospital as a critical facilitator [20]. Similarly, Bilal et al. highlighted the need for support at multiple levels, including family and community [19]. However, the present study adds nuance by demonstrating that such support may also impose financial and emotional strain on spouses, a dimension that is less emphasized in previous literature.

Cultural barriers, such as myths surrounding preterm birth or resistance from fathers, have been reported in Côte d’Ivoire and eastern Uganda [17,18], but were less present in this study. Instead, participants more frequently described unfamiliarity with KMC and practical challenges in adapting the method, especially among twin mothers. This highlights a potential gap in existing guidelines and education, which may not adequately address more complex caregiving situations. Additionally, while previous studies have emphasized healthcare worker motivation as a key facilitator [18,19], participants in this study generally described healthcare workers as supportive, though constrained by systemic limitations.

### Strengths and limitations

A key strength of this study is that it was conducted in a setting where KMC has already been implemented, allowing for insights that are highly context-specific and with direct practical relevance. We used qualitative semi-structured interviews, which enabled the investigation of a broad range of topics while allowing participants to express their experiences in depth, including emotional and contextual aspects relevant to the study aims. The questionnaire was pre-tested and revised, and interviews were conducted. The presence of a local, experienced interviewer likely facilitated trust, reduced language and cultural barriers, and supported accurate transcription and translation.

However, several limitations must be considered. As an inductive qualitative study conducted at a single site with a limited sample, the findings are not generalizable. Selection bias may have occurred, as participant recruitment partly relied on voluntary participation, potentially excluding mothers with more negative experiences. Social desirability bias is also possible, as participants may have been hesitant to express criticism due to dependence on healthcare staff, and the presence of a student observer may have influenced responses. Furthermore, the absence of perspectives from healthcare workers and other family members besides mothers limits the breadth of insights, particularly regarding barriers among mothers who decline or discontinue KMC. The translation and interpretation processes may have resulted in loss of nuances; however, reflexivity was maintained as the interviewer was engaged in the analysis and interpretation of the result. Because mothers who declined or discontinued KMC before the five-day threshold were not captured in this study, this introduces selection bias, as not all women who were admitted to KMC were included, and it is likely that the barriers and dissatisfaction reported understate the difficulties experienced by the wider population of mothers offered KMC at MRRH. However, the aim of the study was to see the adequacy of the KMC in the hospital and the barriers and facilitators for implementation of KMC. To assess the implementation barriers and facilitators for KMC in the hospital, we hypothesized that the women need to stay in the hospital for an adequate time point to understand the routine of the hospital and experience the care.

## Conclusion

Specifically, KMC implementation in MRRH is shaped by multiple interconnected factors across the health system, individual, and socio-economic domains. While mothers generally perceive KMC as beneficial, its consistent practice is influenced by access to resources, quality of education and counselling, financial constraints, and the availability of social support. These factors interact dynamically, where limitations in one area may be partially offset by strengths in another, resulting in implementation that is feasible but fragile. Overall, these findings support the broader literature in demonstrating that KMC implementation is influenced by a complex interplay of health system capacity, socioeconomic conditions, and individual experiences. The consistency of barriers across settings suggests that improving KMC uptake will require not only strengthening clinical services but also addressing structural and contextual determinants, including education, resource availability, and social support systems. Interventions targeting education, infrastructure, and material support, alongside strengthening family and community involvement, may enhance adherence and sustainability of KMC practice.

## Supplementary Material

COREQ checklist.docx

## Data Availability

The transcript can be made available on request.
